# ATP-Mediated Compositional Change in Peripheral Myelin Membranes: A Comparative Raman Spectroscopy and Time-Of-Flight Secondary Ion Mass Spectrometry Study

**DOI:** 10.1371/journal.pone.0142084

**Published:** 2015-11-06

**Authors:** Nikolay Kutuzov, Alexander Gulin, Vladimir Lyaskovskiy, Victor Nadtochenko, Georgy Maksimov

**Affiliations:** 1 Biophysics Department, Biological Faculty, Moscow State University, Leninskie gory 1/12, Moscow, Russian Federation, 119991; 2 N.N. Semenov Institute of Chemical Physics, RAS, Kosigin str. 4, Moscow, Russian Federation, 119991; 3 Chemistry Faculty, Moscow State University, Leninskie Gory 1–3, Moscow, Russian Federation, 119991; 4 All-Russian Research Institute for Optical and Physical Measurements, Ozernaya 46, Moscow, Russian Federation, 119361; 5 Moscow Institute of Physics and Technology State University, Institutskiy per. 9, Dolgoprudny, Moscow Region, Russian Federation, 141700; 6 Institute of Problems of Chemical Physics RAS, Academician Semenov avenue 1, Chernogolovka, Moscow region, Russian Federation, 142432; University of the Pacific, UNITED STATES

## Abstract

In the present paper we addressed a mechanism of the myelin reorganization initiated by extracellular ATP and adenosine in sciatic nerves of the frog *Rana temporaria*. In combination with Raman microspectroscopy, allowing noninvasive live-cell measurements, we employed time-of-flight secondary ion mass spectrometry (TOF-SIMS) to follow the underlying changes in chemical composition of myelin membranes triggered by the purinergic agents. The simultaneous increase in lipid ordering degree, decrease in membrane fluidity and the degree of fatty acid unsaturation were induced by both ATP and adenosine. Mass spectrometry measurements revealed that ATP administration also led to the marked elevation of membrane cholesterol and decrease of phosphotidylcholine amounts. Vesicular lipid transport pathways are considered as possible mechanisms of compositional and structural changes of myelin.

## 1 Introduction

ATP and adenosine are now well established not only as key regulators of cell metabolism and homeostasis but also as compounds forming a sophisticated regulatory network, called purinergic signalling (PS) [1]. The field of PS in the nervous system is rapidly expanding and there are plenty of comprehensive reviews available [2–4]. Here we will focus our attention on the relation of purinergic system to a Schwann cell (SC)—myelinating glial cell in peripheral nervous system (PNS).

The expression of various types of purinergic receptors has been identified in SCs, and it is now well established that both ATP and adenosine are necessary for their development and functioning [1, 5]. The basic molecular events which start with a purinergic receptor activation and lead to the second messenger system activation have been studied in detail. On the other hand, it is not usually considered that there are purinergic pathways the main purpose of which is to alter the cell lipid membrane properties. If we assume that such mechanisms do exist, then in case of a SC it can alter the myelin properties which in turn can influence the nerve cell excitability. Therefore such mechanism can provide additional tools for the purinergic regulatory system to influence the nerve cell activity.

In a recent work Shin et al. demonstrated that extracellular ATP can inhibit myelin degradation of sciatic nerves with induced Wallerian degeneration [6]. This work is a concrete example of how ATP-activated pathway can evoke a reorganisation of peripheral myelin, however the exact mechanism is unclear. Another example of a possible role of ATP in myelin restructuring comes from the previous studies of authors where an administration of extracellular ATP led to the structural changes in myelin lipids [7]. The underlying rationale of the present work is to provide an evidence that ATP can not only affect conformational and ordering properties of myelin lipids, but also change its chemical composition. This result, to our opinion, indicates that PS is more versatile than it is assumed and provides us with an argument towards the discussed above myelin-dependent pathway of ATP in peripheral nerves.

In the present work we aimed at measuring the myelin properties after the administration of ATP using several experimental approaches. Raman spectroscopy permits exploration of untreated samples and has great capability to study membrane fluidity, conformational and orientational ordering of lipids and saturation degree of fatty acid chains [8, 9]. However it is usually impossible to directly analyze chemical composition of a sample and provide information about a certain molecule of interest, especially in studies of complex biological objects.

Mass spectrometry (MS) on other hand is a highly invasive technique requiring operations in vacuum conditions which gives direct information of molecular content of a sample. Researchers have already envisioned wide variety of MS applications in studies of biological objects [10, 11]. MS was employed to investigate cell lipid composition in nervous system under various physiological and pathophysiological conditions [12–14]. In a review paper addressing the lipid metabolism in myelinating glial cells Chrast et al emphasized that the properties of myelin significantly depend on its chemical composition, therefore MS data can yield important details needed for the correct interpretation of the experimental results [15].

We proposed that the combination of these two methods would allow to perform live cell measurements to study the overall effect of ATP and adenosine on physicochemical properties of myelin, as well as provide detailed lipid composition analysis using fixed cells. Using these approaches we were able to show that both ATP and adenosine induced change in lipid ordering, membrane fluidity and fatty acids unsaturation degree. Cholesterol and phosphatidylcholine were identified as possible key mediators of the ATP-induced myelin membranes restructuring.

## 2 Materials and methods

### 2.1 Chemicals

Ringer buffer saline (pH = 7.4) was prepared using the following reagents: NaCl (Sigma) 100 mM, KCl (Sigma) 2 mM, CaCl_2_ (Sigma) 1.08 mM, HEPES (Sigma) 10 mM. ATP, adenosine, and MBCD (Methyl-*β*-cyclodextrin) were purchased from Sigma as powders and dissolved in Ringer buffer at the beginning of experiments. Cholesterol (Sigma) and L-*α*-Phosphatidylcholine from egg yolk (Sigma) were dissolved in ethanol and used for preparation of lipid films.

### 2.2 Animal preparation

All experiments on animals (frog *Rana temporaria*) were carried out in accordance with the animal care regulations of the M.V. Lomonosov Moscow State University. The protocol was approved by the Bioethics Committee of the Faculty of Biology, M.V. Lomonosov Moscow State University (reference number: 17). Prior to experiments male frogs were maintained in containers with some water at +5 C without feeding. Animals were anesthetized using solution of propofol (50 mg/kg) administered intraceolomically, which ensured the deep anesthesia condition prior to euthanasia. Anesthetized animals were decapitated with the following pithing of the brain.

The sciatic nerves were dissected and, ligated at both ends, immersed into Ringer’s buffer solution for a half an hour. Single nerve fascicles were desheated by removing blood vessels, fat, epineurium, and perineurium. Experiments were conducted at room temperature. Recording chambers used for spectroscopic measurements were filled with Ringer’s saline.

### 2.3 Raman spectra recordings

Spectral recordings and Raman imaging of single nerve fibers were performed using confocal Raman mode of the Witec alpha 300 near-field microscope (Germany) with a Nd:YAG laser (532 nm). Typical output power and exposure time for spectral measurements were 0.5 mW and 20 sec respectively. Linearly polarized light was focused through the upright 50x Zeiss objective with *NA* = 0.8. Since the Raman signal from myelin is highly anisotropic it is important to define polarization direction of laser beam in respect to the nerve fiber axis [16]. We chose to collect Raman spectra with polarization of laser parallel to the nerve fiber. In this case we had optimal intensity of both lipid and carotenoid bands. Raman spectra background subtraction was performed using Witec Project software, which employs polynomial fit of a spectral baseline. We used second order polynomials both in 1100–1800 cm^−1^ and 2700–3100 cm^−1^ spectral range.

### 2.4 Fixation of nerve fibers

Small nerve bundles were exposed to 500 *μ*l of either ATP or adenosine solutions for 20 minutes. Next they were used for Raman spectra measurements or fixated for 40 minutes in glutaraldehyde solution. The time of glutaraldehyde exposure was chosen based on the microscopic evaluation of the preservation of fixed fibers morphology. We used 2% glutaraldehyde solution for the fixation, following experimental protocols described elsewhere [17, 18]. After the fixation nerves were rinsed in buffer solution for multiple times to wash out glutaraldehyde residues. Finally, thin nerve bundles were teased and placed on the silicon wafer surface to dry.

### 2.5 TOF-SIMS analysis

A ION-TOF ToF-SIMS 5 mass spectrometer was used with a pulsed primary beam of focused 30 keV Bi^+^_3_ ions. The primary ion current, measured by Faraday cup, was 0.5 pA corresponding to the primary ion dose density ∼ 2.5 × 10^11^ ions/cm^2^, which was below the static SIMS limit. Secondary ions were postaccelerated to 10 keV before hitting the detector. The area used for analysis was 500 × 500 *μ*m^2^ (128 × 128 pixels). A low-energy electron flood gun was activated to avoid charging effect. All spectra were recorded in both positive and negative ion modes. Ion yields were calculated as an intensity of the corresponding peak of interest normalized to the total ion count amount.

### 2.6 Lipid films preparation

To verify TOF-SIMS results and investigate the contribution of the matrix effect (described below) we prepared the following solutions of lipids in ethanol:

1. L-*α*-Phosphatidylcholine from egg yolk (500 *μ*M)
2. Cholesterol (500 *μ*M)
3. L-*α*-Phosphatidylcholine from egg yolk and cholesterol mixture (500 *μ*M/500 *μ*M)
4. L-*α*-Phosphatidylcholine from egg yolk and ATP mixture (500 *μ*M/2 mM)
5. Cholesterol and ATP mixture (500 *μ*M/2 mM)

A probe of 25 *μ*L of each lipid solution was deposited on a silicon wafer and then centrifuged. Obtained dried films were used for TOF-SIMS measurements.

### 2.7 Statistics

Raman spectra measurements were performed on 34 pairs of nerve fibers. For MS measurements we used 23 pair of fixed nerve samples. Error bars in histograms were calculated as the standard deviations from the mean values. Student’s T-test was employed to test the statistical significance of the difference between experimental and control data.

## 3 Results and Discussion

### 3.1 Experimental protocol considerations

First of all we selected the concentration range of extracellular ATP and adenosine which we used for cells treatment. Taking into account previous studies of Shin we chose a similar concentration range (0.25 mM and 1 mM) of ATP and adenosine [6]. An important question arises on whether these values are relevant for normal cell physiology. Most of ATP and adenosine purinergic receptors exhibit their activity in approximately micromolar (or lower) concentration range [3, 19]. An important exception is *P*2*X*7 receptor which activates at almost millimolar concentrations [20]. However it doesn’t imply that administration of millimolar solution of ATP is relevant only to this receptor subtype.

Wewers and Sarkar discussed several important issues, associated with an estimation of local concentration of compounds inside various tissues [21]. In tissues with a highly complicated morphology and diffusion barriers the use of a single quantity like average extracellular concentration can be inaccurate. The release and accumulation of substances in a small compartment with restricted diffusion can lead to high local concentrations. Sciatic nerves have a complicated morphology with oriented nerve bundles and blood vessels all covered with connective tissue layers. This tissue exhibit diffusion anisotropy and hence we should be aware of the described above phenomena [22]. In this sense we believe that used ATP and adenosine concentrations are applicable for myelinated nerve fiber physiology studies.

In our experiments we observed changes in myelin membrane properties (via Raman spectroscopy) and its chemical composition (via mass spectrometry) after 20 minutes of treatment with a compound of interest. More prolonged incubation time can result in the start of nerve fiber degeneration. On the other hand it was shown previously that shorter incubation times (order of few minutes) were shown not to induce any changes in the internodal region of myelin [7]. Taking into account that a myelinated nerve fiber consists approximately only of internodal regions with very small contribution of Nodes of Ranvier and paranodal regions, the processes attributed to the overall myelin reorganisation should be detectable along the whole fiber length.

Taking together, used concentration range and incubation time provided us with optimal balance between the effect magnitude and preservation of nerve fibers condition during the experiment.

### 3.2 Raman spectroscopy of nerve fibers

**Fig 1:**
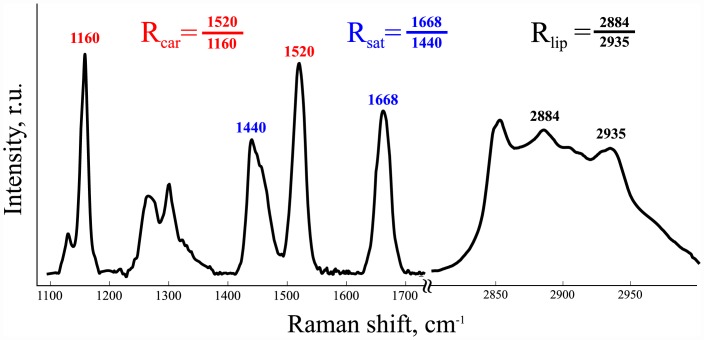
Myelin Raman spectra with main peaks and intensity ratios used for calculations.

Typical Raman spectra of a nerve fiber consists of carotenoid and lipid bands (Fig 1), assignment of which was established previously [16]. Carotenoid Raman bands include 1160 cm^−1^ and 1520 cm^−1^ peaks corresponding to -C–C- and -C = C- band vibrations [23, 24]. Peaks located near 1440 cm^−1^ and 1668 cm^−1^ are attributed to CH_2_ bend and -C = C- stretch modes of fatty acids [25]. Finally, 2884 cm^−1^ and 2935 cm^−1^ can be assigned respectively to asymmetric methylene stretching vibration and symmetric stretching of terminal methyl groups [26].

Combining Raman intensities into various ratios it is possible to calculate quantities which are sensitive to lipid ordering degree and unsaturation of fatty acid chains. Ratios 1520/1160 and 2884/2935 are increased in parallel with an increase of the lipid chain ordering degree and decrease of membrane fluidity [8, 16]. We will further designate them as *R*_*car*_ (carotenoid) and *R*_*lip*_ (lipid). Decrease of a 1668/1440 ratio reflects the increased degree of fatty acid chains saturation, and we will refer to it as *R*_*sat*_[25].

**Fig 2:**
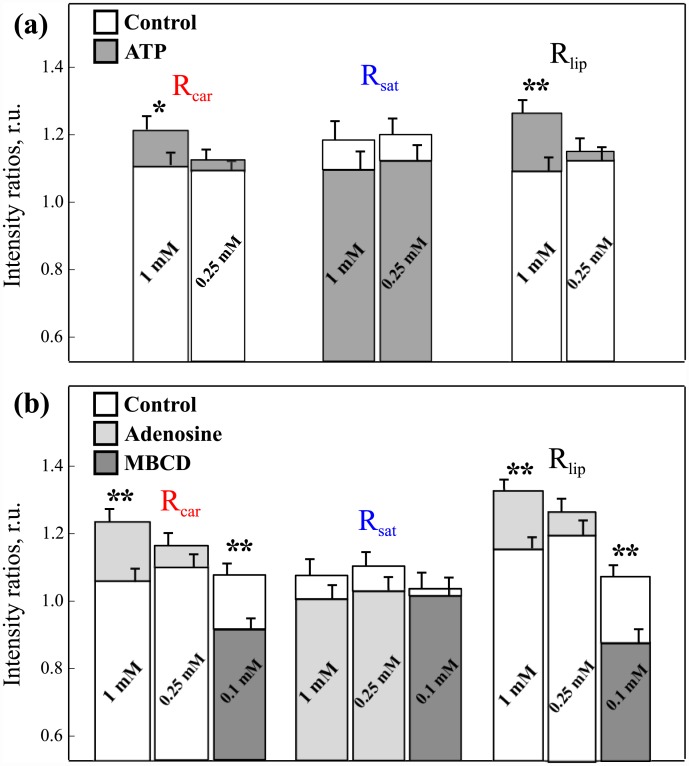
The comparison of Raman intensity ratios after ATP, adenosine and MBCD administration. The corresponding concentrations are shown on histogram bars. Asterisks * and ** denote statistic *p* < 0.05 and *p* < 0.001 respectively.

The administration of both ATP and adenosine in concentration of 1 mM resulted in increased ordering degree of lipids and membrane viscosity, which is clear from the elevation of *R*_*car*_ and *R*_*lip*_ ratios. Oppositely, as shown in Fig 2(a) and 2(b), *R*_*sat*_ was decreased implying the elevated amount of saturated fatty acids. When we lowered the concentration of ATP and adenosine down to 0.25 mM we saw no significant differences in any ratios Fig 2(a) and 2(b).

Both ATP and adenosine can affect cell functioning in a variety of ways, so we will briefly describe some of them, which can be of relevance to our results. Purinergic compounds were shown to activate calcium signaling in nerve cells which can in turn trigger various other pathways. For instance, Tuck and Cavalli showed that the process of membrane resealing after nerve injury is mediated by endogenous vesicles and is highly dependent on intra-axonal level of calcium [27]. Different pathways including activation of P2 and P1 purinergic receptors can result in intracellular calcium elevation. The very basic mechanism for ATP (or ADP) induced calcium influx is via P2 receptors [1].

Another conceptually distinct pathway is the P1 metabotropic receptor activation. Adenosine is a main ligand for P1 receptors, although AMP was also recognized as a ligand for the A1 receptor subtype [28]. Activation of P1 receptors can start second messenger cascades, which lead to calcium mobilization from endoplasmic retuculum [29, 30]. After administration of ATP into the bathing solution it becomes the substrate for ectonucleotidases—enzymes, located at the outer cell surface. The step by step process of ATP breakdown involves sequential diphosphorylation and yields ADP, AMP and finally adenosine [31]. Therefore, both ATP and adenosine treatment can result in activation of P1 receptors.

Consequently, there is a plenty of possibilities of how the ATP or adenosine can interact with a nerve cell in PNS. Purinergic receptors are the likely candidates, however, finding out the responsible receptor type requires a detailed inhibitor analysis.

### 3.3 TOF-SIMS mass spectrometry of nerve fibers

TOF-SIMS allows parallel detection of multiple species without any sample treatment except fixation for vacuum conditions. However, there are several important limitations of the method one needs to mention. First, poor high mass sensitivity did not allow us to carry out peptides and proteins analysis. Second, matrix effect (discussed below) drastically complicates data quantification. [32]. Nevertheless, comparison with control samples allows to distinguish the difference in chemical composition and perform semi-quantitative data interpretation [33].

**Fig 3:**
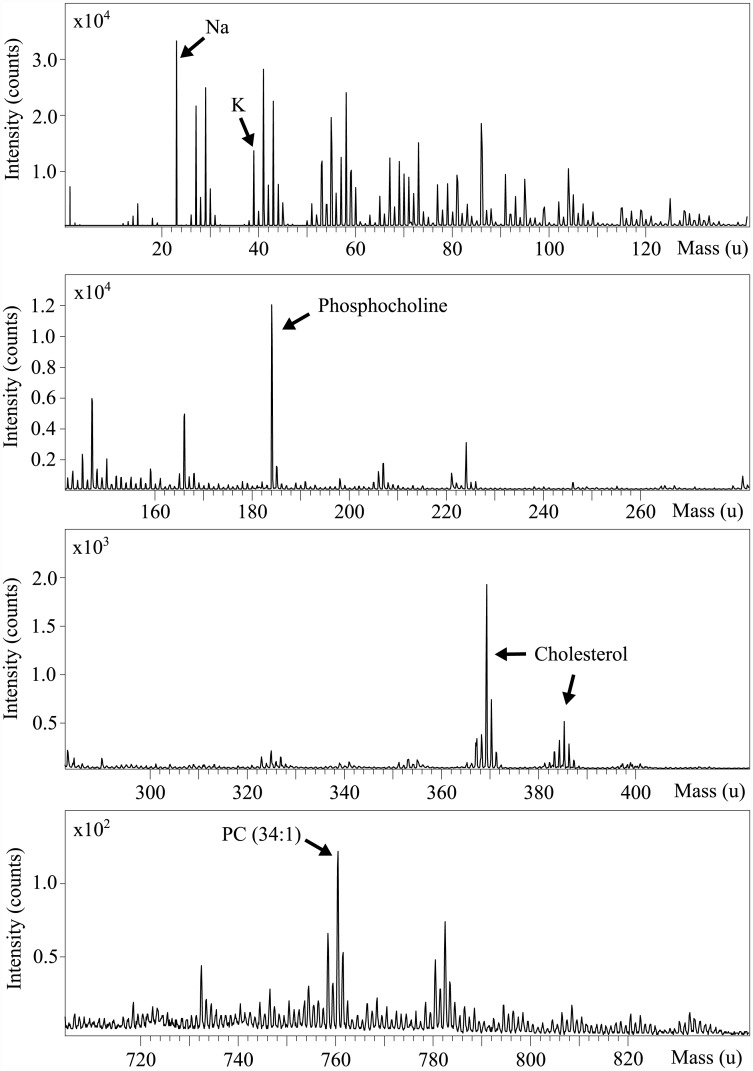
TOF-SIMS spectra of myelin.

We employed the TOF-SIMS to study the effect of ATP and adenosine on the lipid composition of myelin. Nerves incubated with ATP or adenosine were fixed and analyzed. Fig 3 illustrates a representative example for the positive ion mass spectrum of myelin. Due to the specifics of the currently employed method, which are described elsewhere [32, 34], detectable mass range is limited to several thousand of Daltons. Thus lipids, metals and various metabolites can be analyzed using this setup.

Ion yields of several peaks of interest were evaluated and compared with control. Sodium (*m*/*z* = 22.99) and potassium (*m*/*z* = 38.96) ions are easily identified in the measured spectra. The most intense lipid peaks are phosphocholine (*m*/*z* = 184.07), which is a head group (a fragment ion) of phosphatidylcholines (PC) and sphingomyelines, cholesterol (*m*/*z* = 369.35 and 385.35) and a phosphatidylcholine (34: 1) molecular ion (*m*/*z* = 760.60).

**Fig 4:**
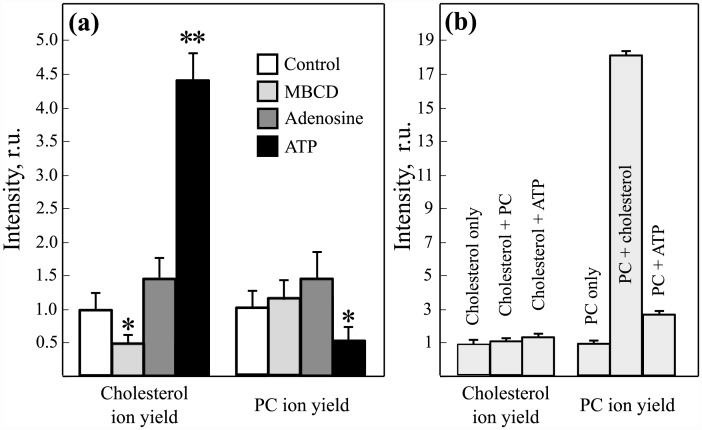
Differences in relative amounts of cholesterol and PC in nerve fibers treated with MBCD, ATP and adenosine (a). Estimation of the matrix effect by measuring cholesterol and PC ion yield in lipid films of different composition (b). Asterisks * and ** denote statistic *p* < 0.05 and *p* < 0.001 respectively.

Sodium level signal change was difficult to estimate due to detector saturation effect. Cholesterol and phosphatidylcholine (34:1) ion signals level changes are shown in Fig 4(a). These molecular ions exhibited the most pronounced changes, therefore we used them for further analysis. Results showed that administration of ATP significantly enhanced cholesterol signal in approximately 4.5 times relative to the control sample and decreased PC ion yield by about 40%.

### 3.4 Model experiments to estimate the matrix effect

Matrix effect is a complicated factor that must be considered in TOF-SIMS analysis [32, 35, 36]. It is based on the fact that cholesterol tends to form deprotonated molecular ion [M − H]^+^ which could be a source of additional protons for [M + H]^+^ ion species, particularly PCs. Caution is therefore needed in the interpretation of MS data.

To estimate the possible effect of ATP on cholesterol and phosphatidylcholine (34:1) ion yields, lipid films were prepared and analyzed using TOF-SIMS. Control samples were prepared from either cholesterol or PC. Two films were made using cholesterol or PC mixture with ATP. Finally last film was made from PC and cholesterol mixture, since cholesterol can affect PC ion yield. Results of MS measurements are represented in Fig 4(b).

As it has been suggested cholesterol administration led to the significant PC signal enhancement. The presence of ATP also increased PC ion yield. According to this data one may conclude that genuine PC signal level diminution is likely to be more than 40%. In contrast, cholesterol didn’t show any considerable change in ion yield due to matrix effect, which means that the enhancement factor of ∼ 4.5 is caused by changes in chemical composition of myelin membranes. Collectively these observations indicate that treatment with 1 mM ATP and not adenosine induced the significant elevation of cholesterol and decrease of PC amount in myelin membranes.

### 3.5 Raman spectroscopy measurements of myelin membranes with extracted cholesterol

In order to correlate the data from Raman and TOF-SIMS measurements we studied how the change in cholesterol can influence the Raman ratios. We applied 0.1 mM MBCD to the nerve fibers and collected spectra after 20 min of incubation. MBCD is a widely used compound for cholesterol extraction from lipid membranes [37]. From Fig 2(b) it clear that MBCD administration resulted in decrease of both *R*_*car*_ and *R*_*lip*_ ratios and had no effect on *R*_*sat*_. Hence the elevation of membrane cholesterol is likely to increase the *R*_*car*_ and *R*_*lip*_ ratios, which is exactly what we observe in experiment. Therefore we believe that changes in membrane fluidity and lipid ordering degree induced by ATP can be caused by the elevation of membrane cholesterol. The decrease of 1668/1440 Raman ratio indicates the increase of fatty acid saturation degree which can have the similar effect on membrane fluidity.

### 3.6 Conclusions

Summarizing the obtained results, we can conclude that ATP treatment of isolated peripheral nerve fibers led to the increased cholesterol and decreased PC ion yield in myelin membranes. From Raman spectra it is clear that adenosine has the same effect as ATP of equal concentration (Fig 2). However, we saw no significant differences in cholesterol or PC ion yields from MS data. Adenosine by itself could be not as potent as ATP and therefore produced only a modest cholesterol increase which was intense enough to measure via Raman microscopy but not using MS.

In TOF-SIMS analysis using adequate sample preparation technique and the same experiment conditions for both analyzed and control samples do not guarantee correct correlation between secondary ion signal and changes in chemical composition due to the matrix effect. Here we made an attempt to estimate influence that might be caused by ATP administration or cholesterol enrichment. We found that the observable differences in ATP-treated samples are much more likely to be caused by the actual changes in the amount of cholesterol and PC and not by modulation of their ion yields by ATP.

From the obtained data it is challenging to propose a concrete interpretation (cellular mechanism), which we believe requires further investigation by combining biophysical methods for myelin structure and composition analysis as well as biochemical assays to provide an insight into the intracellular pathways.

From the general point of view, an elevation of the cholesterol level can be due to its synthesis or redistribution (within different cellular compartments). Several research papers investigated the characteristic times needed for cholesterol synthesis and incorporation into plasma membranes [38, 39]. The measured value is usually in the order of tens of minutes which is applicable to our case, and thus we are not able to suggest whether we are dealing with already existed or newly synthesized cholesterol. In regards to the possible biological mechanism we would like to mention a work by Shin et al. on the effect of ATP on the demyelination process in nerve fibers. They showed that ATP can activate lysosomal exocytosis, which delayed the degradation of myelin [6]. The change in the cholesterol amount in myelin based on the its transfer from the endogenous vesicles, in our opinion, is one of the good initial guesses in a search of the underlying mechanism.

We believe that described results are important for the beginning of understanding the new mechanisms of peripheral myelin structure regulation, by means of the change in lipid composition. Finally it is important to mention the fact that ATP is released into extracellular space during nerve injury [40]. In this case compositional change of myelin membranes can therefore be a stabilizing tool to enhance cell integrity or be a part of a more general ATP–mediated regulatory pathway.

## Supporting Information

S1 Dataset Control Raman spectra for the data in Fig 2. (ZIP)

S2 Dataset Raman spectra from ATP experiments supporting the data in Fig 2. (ZIP)

S3 Dataset Raman spectra from adenosine experiments supporting the data in Fig 2. (ZIP)

S4 Dataset Raman spectra from MBCD experiments supporting the data in Fig 2. (ZIP)

S5 Dataset Mass spectra from ATP experiments supporting the data in Fig 4. (ZIP)

S6 Dataset Mass spectra from adenosine experiments supporting the data in Fig 4. (ZIP)

S7 Dataset Mass spectra from MBCD experiments supporting the data in Fig 4. (ZIP)
